# Assessing severity of anaphylaxis: a data-driven comparison of 23 instruments

**DOI:** 10.1186/s13601-018-0215-x

**Published:** 2018-08-01

**Authors:** Esben Eller, Antonella Muraro, Ronald Dahl, Charlotte Gotthard Mortz, Carsten Bindslev-Jensen

**Affiliations:** 1 Odense Research Center for Anaphylaxis (ORCA), Department of Dermatology and Allergy Center, Odense University Hospital, Odense, Denmark; 2 Food Allergy Referral Centre – Veneto Region, Department of Women and Child Health, Padua University Hospital, Padua, Italy; 3 GSK, Brentford, Middlesex, UK

**Keywords:** Severity assessment, Anaphylaxis, Severity comparison, Kappa statistics

## Abstract

**Backgroud:**

The severity of an allergic reaction can range from mild local symptoms to anaphylactic shock. To score this, a number of instruments have been developed, although heterogeneous in design and purpose. Severity scoring algorithms are therefore difficult to compare, but are frequently used beyond their initial purpose. Our objective was to compare the most used severity scoring instruments by a data-driven approach on both milder reactions and anaphylaxis.

**Methods:**

All positive challenges to foods or drugs (n = 2828) including anaphylaxis (n = 616) at Odense University Hospital, Denmark from 1998 to 2016 were included and severity was scored according to Sampson5. Based on recommendations from an expert group, the symptoms and values from Sampson5 were for all reactions and anaphylaxis only translated and compared by kappa statistics with 22 instruments, ranging from 3 to 6 steps.

**Results:**

For milder reactions, there was a significant correlation between the number of steps in an instrument and the number of challenges that could be translated, whereas all instruments were good to identify food anaphylaxis. Some instruments scored reactions more severely than Sampson5, other scored them milder and some scored food and drug challenges differently. Instruments for hymenoptera reactions were difficult to apply on food and drug reactions, and thus distributed severity differently. Algorithms hampered the translation between instruments, and 7 instruments were poor concerning drug anaphylaxis, including the only instrument developed specifically for drug reactions.

**Conclusion:**

The distributions of severity differed between the 23 instruments in both food and drug allergy, and thus rendering translation especially between scoring systems with 3 and 5 grades difficult. Fine-graded and simple instruments are preferred for comparison especially among milder reactions, and instruments applied to non-intended situations may not reflect a true severity picture.

## Background

The severity of an allergic reaction can range from subjective local symptoms to lethal anaphylactic shock. Dosage, individual threshold, route of exposure, type of allergen, age, comorbidity and involvement of facilitators can influence the severity, and this combined with the progression of symptoms and the ambiguous definition of anaphylaxis [1], makes severity difficult to capture. Furthermore, the settings in which the reaction occurs are far from comparable, ranging from accidental exposure in an unknown environment to controlled challenges in a highly specialized clinical setting.

Multiple scoring instruments have been developed to assess the overall severity of an allergic reaction, elicited either by foods [2–9], drugs [10] or hymenoptera stings [11–14]. All instruments cover the whole spectrum of symptoms and signs, and several are using the term anaphylaxis to describe their scoring algorithm, although it is evident that non-anaphylactic milder symptoms neither fulfill the WAO [1, 15] nor the new ICD-11 [16] criteria. Many of these instruments are today applied beyond their initial purpose, whereas others have been adopted to span multiple causes [17–21]. Data-driven instruments are scarce [5, 9, 22] and the majority of tools are designed empirically for data collection in emergency rooms (ER) or intensive care units (ICU) [10, 13, 14, 17–20], in clinical trials (CT) [4, 6, 7, 12], or based on consensus reports, theoretical reviews, position papers, or national guidelines [8, 21, 23–27]. All instruments have organ-specific outcomes, dividing symptoms according to their anatomical origin, i.e. skin, respiratory, gastro-intestinal (GI), cardio-vascular (CV) or neurological symptoms. Some use a detailed predefined “symptom list, ranging from a binary form of “present/not present” to detailed grading of specific symptoms, e.g. urticaria, into mild/local or severe/generalized. Others use more general ‘catch-all’ symptoms from a specific organ, e.g. all symptoms related to the “GI tract”. All operate with an ordinal scale spanning over 3–6 incomparable steps, where the overall severity either is defined by the highest numerical value, i.e. most severe symptoms [7, 8, 10, 11, 14, 17, 21, 23–25], relative allergen exposure [6, 18], milder symptoms obligate for severity progressing [4], fulfillment of “2-or-more” [13], summation of symptoms to get severity [12, 28] or related to number of organs involved [2, 5, 9, 27, 29].

The ideal severity assessing instrument should span all ages (children/adolescents/adults), all allergens (foods, insects, drugs), all exposure circumstances (exercise and other co-factors, injection, inhalation, oral intake etc.) and cope with the whole spectrum of symptoms. This instrument should work as a measuring tool for patients and clinicians, applicable in primary care, ER/ICU, research projects, and combining existing instruments, i.e. being retrograde compatible. A prospective comparison between existing instruments, applied in situ at the same exposure or on the same patient, would be ideal, but this is time consuming, raises ethical dilemmas on when to treat with adrenalin due to ambiguous stop-criteria and does not solve the issue of precise definition of existing tools. Knowing that titrated challenges are not ideal for addressing severity of anaphylaxis, a retrospective data-driven validation, comparing instruments based on robust clinical challenge-verified data could be the second best option to compare the translatability and distribution of severity between instruments, and could form basis for development of a common standardized instrument.

Our aim was retrospectively to compare the distribution of severity in existing grading instruments, by applying each of them to a well-characterized clinical database covering both anaphylactic and milder reactions, based on the definition of an expert-group within the fields of dermato-allergology, respiratory-allergology and pediatric allergology. Ideally, we would provide a platform for subsequent development of a universal scoring instrument for anaphylactic reactions. This study is neither testing the efficacy of intruments to identify anaphylaxis, nor should it be seen as a literature overview of existing severity assessing instruments, but instead as a comparison of the, to our knowledge, most used instruments.

## Methods

Data, i.e. recorded objective signs and/or subjective symptoms, from all positive food (n = 2382) or drug challenges (n = 466) at the Odense Research Center for Anaphylaxis (ORCA) from January 2001 to January 2016 were consecutively entered into a database and included. Anaphylaxis according to WAO criteria [15] was seen in 22% (535/2382) of the food challenges and 19% (84/446) of the drug challenges. Egg (n = 720), peanut (n = 579), hazelnut (n = 264) and milk (n = 230) were the most frequent food allergens, whereas penicillin accounted for $${\raise0.7ex\hbox{$2$} \!\mathord{\left/ {\vphantom {2 3}}\right.\kern-0pt} \!\lower0.7ex\hbox{$3$}}$$ and non-steroidal, anti-inflammatory, drug (NSAID) for $${\raise0.7ex\hbox{$1$} \!\mathord{\left/ {\vphantom {1 3}}\right.\kern-0pt} \!\lower0.7ex\hbox{$3$}}$$ of the drug challenges (see Table 1).

**Table 1:** Characteristics of included challenges and severity distribution of Sampson5 for foods and drugs challenges

	n	# Allergens	Mean age (years [SD])	Gr. 1	Gr. 2	Gr. 3	Gr. 4	Gr. 5
Total	2848	114	17.6 [18.4]	296 (10%)	1253 (44%)	843 (30%)	416 (14%)	20 (1%)
Foods (anaphylaxis^a^)	2382 (535)	86	11.6 [4.0]	198 (0)	1026 (0)	800 (177)	347 (347)	11 (11)
0–3 years	859	22	2.3 [1.0]	33 (4%)	422 (49%)	285 (33%)	118 (14%)	1 (0%)
4–15 years	990	43	7.8 [3.0]	59 (6%)	384 (39%)	408 (41%)	136 (14%)	3 (0%)
15+ years	533	73	33.9 [14.0]	106 (20%)	220 (41%)	107 (20%)	93 (17%)	7 (1%)
Drugs (anaphylaxis^a^)	446 (84)	28	43.8 [17.3]	98 (0)	227 (0)	43 (6)	69 (69)	9 (9)
Antibiotics	285	21	44.6 [17.7]	79 (28%)	154 (54%)	24 (8%)	21 (7%)	7 (2%)
NSAID	143	7	41.1 [16.8]	15 (10%)	69 (48%)	16 (11%)	41 (29%)	2 (1%)

^a^According to WAO [15]

**Fig. 1:**
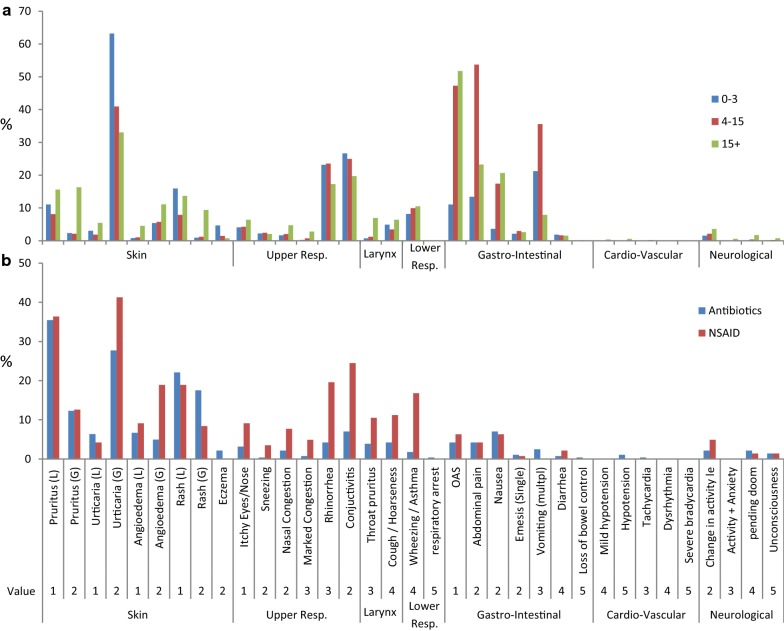
Percentage of challenges with recorded specific symptoms after foods (**a**) and drugs (**b**). Food challenges are age-divided into 0–3, 4–15 and 15+ years, whereas drug-challenges are divided into antibiotics and NSAID’s

The most frequently recorded symptoms after food challenges were urticaria (47%), oral allergy syndrome (OAS) (35%), abdominal pain (32%), conjunctivitis (24%), vomiting (24%) and rhinorrhea (22%). For drug challenges, skin symptoms were predominant; either localized or generalized pruritus (47%), urticaria (36%), rash (35%) or angioedema (17%) (Fig. 1).

The overall severity (Table 1) was based on Sampson5 [8] with an addition of 3 milder symptoms including upper airways and/or eyes (itchy eyes/nose, conjunctivitis) and abdominal pain. Patients challenged to food were significantly younger than patients challenged to drugs (p < 0.001), and food-challenges were therefore subdivided into 3 age-categories (0–3, 4–15, and + 15 years). There was a significant difference between the distribution of severity grading between the 3 age-classes (p < 0.001). Children in the 0–3 years group rarely had subjective symptoms, such as OAS, abdominal pain or nausea, whereas 63% of them had urticaria and/or rhino-conjunctivitis, resulting in often having their challenge stopped after a grade 2 reaction (p < 0.001), compared to older age groups (4–15 years/+ 15 years). Group + 15 years was even more polarized in its severity grading, i.e. often significantly (p < 0.001) milder (gr. 1) symptoms, characterized by a generally higher level in subjective skin symptoms and abdominal pain, but also more frequent severe reactions (gr. 4–5) (p < 0.001). This effect disappeared after adjustment for specific allergens (i.e. milk, egg, peanut, hazelnut) and was entirely driven by more severe objective reactions after a challenge with peanuts [a-OR (95% CI) = 1.77 (1.33–2.35)]. In the + 15 years group, food challenges were significantly milder (p = 0.03) than drug challenges. Reactions to NSAID were more severe than antibiotics (p < 0.0001), caused by more frequently respiratory distress, especially laryngeal and lower respiratory symptoms (p < 0.001).

### Translation of symptoms to other instruments

A direct literature-search identifying severity instruments was not feasible, since the majority of instruments were not published as such, but developed as tools for specific use, e.g. to address severity of reactions in allergen immune therapy trials. Included instruments were therefore identified empirically or in relation to the European Academy of Allergy and Clinical Immunology (EAACI) taskforce initiative on Food Allergy and Anaphylaxis [30]. We identified 22 previously published instruments focusing on severity of allergic reactions [2, 4, 6, 7, 9–14, 17–21, 23–29]; however, 2 were excluded for not addressing the overall severity but more listing symptoms [19, 26]. Moreover, the new EAACI taskforce guidelines [30] (newEAACI3), a 3-step organ-specific “catch-all” instrument and the new iFAAM oFASS instrument [5], a 5-step observational instrument (iFAAM5) were included. With Sampson5 [8], a total of 23 instruments were compared (Table 2).

**Table 2:**
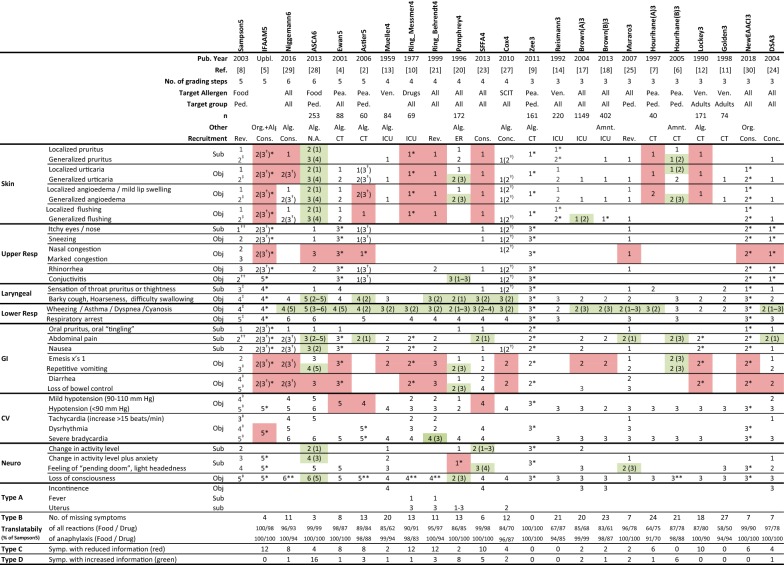
Overview of the 22 included studies, their origin and exact numerical value (1–6) for each listed symptom ordered by organ and appearance in Sampson5

General discrepancies (Type A–D error) between Sampson5 and the comparing instrument are listed below; symptoms missing in Sampson5 (Type A), symptoms missing in the comparing instrument (Type B), symptoms where comparing instrument contained less information than Sampson5 (Type C—symptoms marked in red), and symptoms with increased information in comparing instrument compared to Sampson5 (Type D—marked in green)

*Pea* peanut, *Ven* venom, *Ped* only pediatric, *Alg* inbuilt algorithm, *Amnt* allergen amount depended, *Org* organ specific, *Rev* review, *Cons* consensus, *CT* clinical trial, *ICU* intensive care unit, *ER* emergency room, *GI* gastro-intestinal, *CV* cardio-vascular, *Neuro* neurological, *Resp* respiratory

*Unspecific “catch-all” symptom

**Added variables to reduced number of Type B error among the most severe cases

^†^Translation depending of number of involved organs

^††^Added variables form Sampson5

^‡^Anaphylaxis according to WAO [15]

Numerical values for symptoms in Sampson5 (e.g. value 2 for generalized urticaria) were then retrospectively translated according to recommendations by the expert group into the corresponding value in the comparing instruments (e.g. value 1 in Mueller4 [13]). As illustrated in Table 2, we identified 4 types of systematic translational errors (A–D). Type A errors were symptoms missing in Sampson5, i.e. “incontinence” or “fever”, whereas type B were missing variables in the comparing instrument and therefore untranslatable (i.e. localized urticaria in Mueller4 [13]). Sampson5 includes a total of 23 symptoms from 7 “organs” with a total of 34 possible outcomes. Zee3 [9] was, due to its unspecific “catch-all” structure, the only instrument which embraced all symptoms covered by Sampson5, whereas no other instrument showed complete translatability; the best overlap was 32/34 with ASCI6 [28] and poorest overlap was 8/34 with Golden3 [11]. Type B errors reduced the translatability, i.e. the percentage symptoms translated compared to Sampson5 (Table 2) and additionally led to a systematic discrepancy in those cases, where the most severe symptoms were missing, and thereby determining the overall severity by less severe symptoms; half of the recorded grade 5 reactions were caused by fainting, a symptom missing in 5 instruments [2, 6, 10, 21, 29], resulting either in downgrading to other less severe symptoms or being completely lost in translation. “Unconsciousness” was therefore added to these 5 instruments (marked ** in Table 1), corresponding to the highest numerical value for each system. Other errors were type C, where information was lost, because the comparing instrument contained fewer variables than Sampson5, i.e. “local urticaria” or “generalized urticaria” reduced to “urticaria” (marked in red in Table 1). Finally type D, where the comparing instrument incorporated more information than Sampson5, resulting in a translation based on expert interpretation, i.e. whether “wheeze, asthma, dyspnea, cyanosis” should be translated into “mild wheeze” or “pronounced dyspnea” (green in Table 1). “Catch-all” symptoms, e.g. “all symptoms from GI” were encountered to embrace all possible symptoms for that specific organ. Sampson5 included the lower respiratory symptom “wheeze/asthma/dyspnea/cyanosis”, which was translated into “asthma” in cases with multiple unambiguous translation possibilities, e.g. “wheeze”, “asthma”, or “cyanosis”. Shock and hypotension was defined as systolic blood pressure < 90 mm Hg.

The majority of instruments applied a simple “most severe” symptom to define the overall anaphylaxis severity [7, 10, 11, 14, 17, 18, 21, 23–25, 30], however 11 of the included instruments instead had a built-in algorithm (marked Alg. in Table 2); Ewans5 [4] mandates at least one symptom from grade 1 (localized skin) or grade 2 (generalized skin) plus symptoms from GI/eyes/nose to accomplish gr. 3. For Niggemann6 [29], Astier5 [2], iFAAM5 [5], and Cox4 [27], grade 2 or grade 3 was directly linked to the number of included organs (one vs. multiple organs). Zee3 [9] calculates a none-linear severity index in tertiles, based on involved organs regardless of number of observed symptoms. Mueller4 [13] mandates at least 2 milder symptoms plus the defining symptom to qualify for anaphylaxis > grade 1, whereas Pomphrey4 [20], Lockley3 [12] and ASCA6 [28] included different numerical severity indexes, from which specific symptoms were recalculated to give an overall score. Due to absence of specific symptoms (type B error) for the latter 4 instruments, there was a marked reduction in the number of translatable challenges; e.g. for Mueller4 < 50% fulfilled the 2-or-more criteria. The simple “highest” possible symptom was therefore applied to these four instruments.

### Statistics and translational algorithms

Comparison of severity, age, specific symptoms and type of allergen in Sampson5 was performed with ordinal logistic regression. To compare the distribution of severity between instruments with 3 steps, Sampson5 was reduced into three theoretical grade 3 scales; a scale milder than the original Sampson5 was obtained by merging grade 1 + 2 into 1, grade 3 + 4 into 2 and maintaining grade 5 as a new grade 3 (i.e. grade 1, 2, 3, 4, 5 become 1 + 2, 3 + 4, 5), a scale with similar severity distribution (1 + 2, 3, 4 + 5) and a scale with more severe severity distribution (1, 2 + 3, 4 + 5) than the original Sampson5. Using weighted kappa statistics, all 3-step-instruments [6, 7, 9, 11, 12, 14, 17, 18, 24, 25, 30] were stepwise compared toward these 3 theoretical scales and the best agreement was identified, thereby ordering them into milder, similar or more severe than Sampson5. Similar, four theoretical 4-step-scales were constructed from Sampson5 for comparison between all instruments containing 4 steps [10, 13, 20, 21, 23, 27]. Five-step scales [2, 4, 5, 8] were directly compared to Sampson5, whereas the two instruments containing 6 steps [28, 29] were converted into 6 possible 5-step scales, which then were compared to Sampson5 using weighted kappa statistics. The cumulative distribution function (CDF) for all instruments was plotted against the relative percentage severity of each instrument, i.e. as tertiles, quartiles, quintiles, and sextiles. The Area Under each CDF Curve (AUC) was calculated and the translatability was compared with nonparametric Spearman correlation test. *WAO criteria* [15] *of anaphylaxis were applied to all challenges and 619 challenges fulfilled these* (*see Table* 1). *Challenges identified as anaphylactic were then translated according to previous description and statistical analysis repeated for these.* All calculations were performed in STATA14 SE (Stata Corporation, College Station, TX, USA). *The study was approved by the local board of* Danish Data Protection Agency (license no. 2012-58-0018/journal no. 16/31454).

## Results

Based on symptoms from all 2382 positive food challenges and 446 positive drug challenges, the 22 instruments were translated from Sampson5. Translatability for foods and drugs for all instruments are presented in Table 2. Best translatability was found for Zee3, iFAAM5, ASCA6, SFFA4 and the NewEAACI3 [5, 9, 23, 28, 30], were > 97.5% of all challenges could be translated, whereas only 56% of all challenges could be translated into Golden3 [11]. Mueller4, DSA3, Muraro3, Brown(A)3 and Brown(B)3 [13, 17, 18, 24, 25] were significantly better to translate food challenges than drug challenges, as opposed to Reismann3 and Hourihane(A)3 [7, 14]. There was a significant correlation between the translatability from Sampson5 and the number of steps in the receiving instruments for both foods (r_s_ = 0.57, p < 0.01) and drugs (r_s_ = 0.72 p < 0.005), meaning that instruments with 5 steps less frequently had incomplete translation compared to instruments only containing 3 or 4 steps. Only applying anaphylactic challenges increased the translatability > 90% for all instruments, except 7 instruments on drug anaphylaxis [2, 6, 7, 10, 14, 18, 27]; Ring/Messmer4 criteria [10] only translated 83% of drug anaphylaxis compared to 91%, when milder reactions were included.

**Fig. 2:**
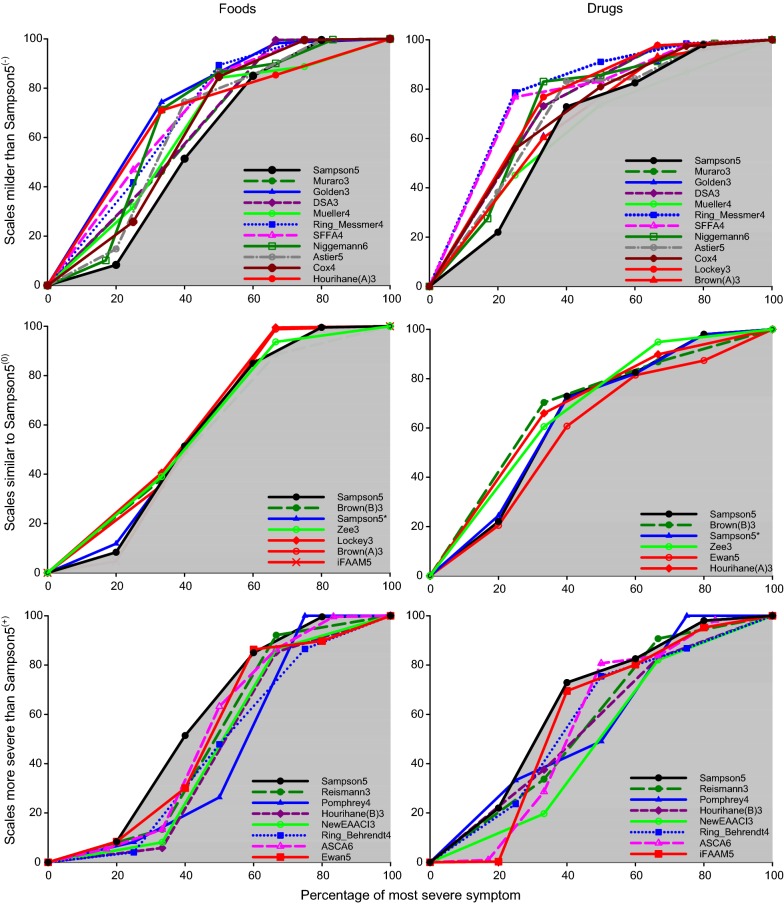
Cumulative distribution function (CDF) of all instruments plotted against percentage of most severe value, and presented for food and drug challenges, respectively. Instruments are divided into the relative shift compared to the Sampson5 (black line) based on kappa statistics. Red lines indicate instruments with different distribution between food and drug challenges. ^(−)^3-step scales with best concordance (highest Kappa values) to Sampson5 recalculated as 1 + 2, 3 + 4, 5. 4-step scales with best concordance (highest Kappa values) to Sampson5 recalculated as 1 + 2, 3, 4, 5. Niggemann6 had best concordance to Sampson5 when recalculated into 1, 2, 3, 4 + 5, 6. ^(0)^3-step scales with best concordance (highest Kappa values) to Sampson5 recalculated as 1 + 2, 3, 4 + 5. ^(+)^3-step scales with best concordance (highest Kappa values) to Sampson5 recalculated as 1, 2 + 3, 4 + 5. 4-step scales with best concordance (highest Kappa values) to Sampson5 recalculated as 1, 2, 3, 4 + 5. ASCA6 had best concordance to Sampson5 as when recalculated into 5-step scale = 1 + 2, 3, 4, 5, 6. *Sampson5 unmodified

The cumulative distribution function (CDF) for all instruments was plotted against the relative percentage severity of each instrument, i.e. the severity in a grade-3 instruments were presented as tertiles (i.e. 33, 66 and 100%) and a grade 5 instruments as quintiles (i.e. 20, 40, 60, 80, and 100%) (Fig. 2). Based on kappa statistics, we could identify three possible scenarios; instruments with left-skewed CDF and thereby overall milder severity-scoring than Sampson5 (Muraro3, Golden3, DSA3, Mueller4, Ring_Messmer4, SFFA4, Cox4, Astier5 and Niggemann6 [2, 10, 11, 13, 23–25, 27, 29]), similar distribution as Sampson5 (Zee3 and BrownB3 [9, 18]) and instruments with a right-skewed CDF and hence a more severe symptom scoring than Sampson5 (Reismann3, HourihaneB3, NewEAACI3, Pomphrey4, Ring_Behrendt4, and ASCA6 [6, 14, 20, 21, 28, 30]). Five instruments (HourihaneA3, Lockey3, BrownA3, Ewan5, and IFAAM5 [4, 5, 7, 12, 17]) showed different distribution on food than drug challenges compared to Sampson5 (red lines in Fig. 2).

**Fig. 3:**
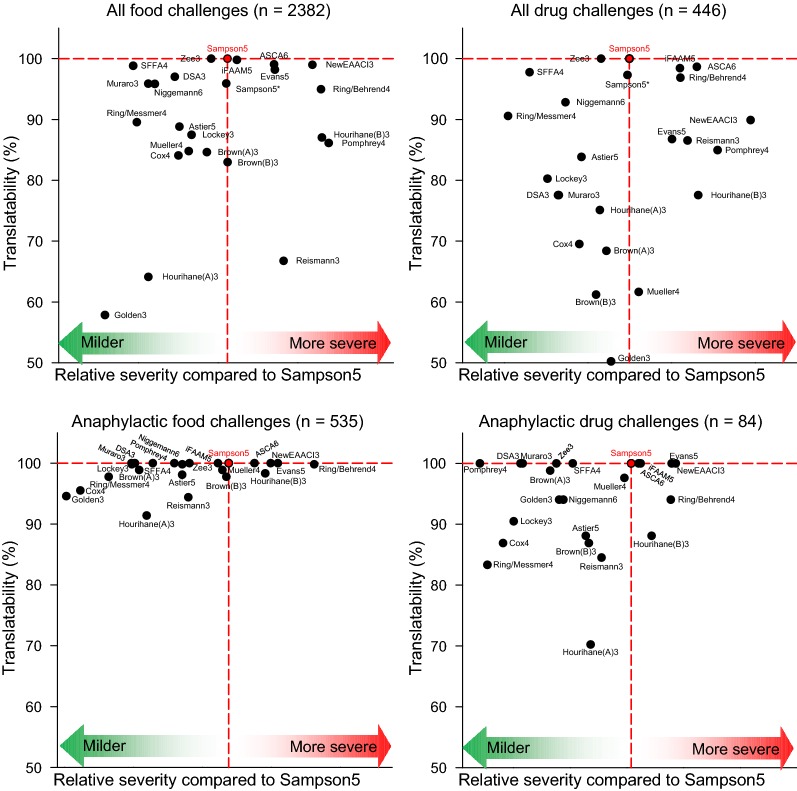
Relative severity and translatability of all instruments compared to Sampson5 for food and drug challenges. The relative severity is presented as 10,000-AUC for each CDF curve, whereas translatability represent the percentage of challenges in Sampson5 to be translated. *Sampson5 unmodified

The area under curve (AUC) for CDF was calculated (for Sampson5 marked in grey in Fig. 2). Corresponding values of translatability (% translated symptoms compared to Sampson5) and AUC for foods and drugs are presented in Fig. 3, both for all symptoms and signs (Sampson grade 1 through 5) and for the 535 anaphylactic food and 84 anaphylactic drug challenges. The relative severity compared to Sampson5 were for most instruments unaffected when only anaphylactic reactions were included; only Reismann3, Pomphrey4, Brown(A)3 and Cox4 [14, 17, 20, 27] distributed food challenges milder, whereas Mueller4 [13] appraise anaphylactic food challenges as more severe. Reismann3 and Pomphrey4 [14, 20] scored drug anaphylaxis milder than non-anaphylactic reactions, indicating that they weighted milder symptoms more, than other instruments.

## Discussion

The aim of this project was to compare existing severity instruments and identifying pros and cons among them, thereby forming a backbone for the development of a future instrument, which ideally should be retrograde compatible. To our knowledge, no study has applied multiple instruments on the same allergic reaction, and this paper is the first data-driven comparison of multiple anaphylaxis severity-scoring instruments, based on challenge-data from more than 12,000 titrated challenges.

The overall heterogeneity between included instruments, i.e. their origin, structure and output was large; some instruments are purposed solely for single allergens, e.g. peanut or bee venom, others developed exclusively for specific populations, i.e. children and some to specific situations, e.g. after immunotherapy trials. The consequent extrapolation of instruments into non-intended situations, lead to discrepancies; instruments developed to cope with hymenoptera reactions [11–14] overall had poor translatability and distributed severity differently compared to Sampson5, but were on the other hand not evaluated in venom anaphylaxis in this study. The only instrument intended on adverse drug reactions (Ring_Messmer4) [10] scored for food challenges milder than Sampson5.

Distributions in severity were different, some instruments overestimated e.g. having more severe reactions than Sampson5 (Ring_Berend4, NewEAACI3 [21, 30]), others underestimated (Muraro3, Mueller4, Ring_Messmer4 [10, 13, 25]), some scored food challenges more severe than drug challenges (Hourihane(A)3, iFAAM5 [5, 7]), and others drug challenges more severe than food challenges (Brown(A)3, Evan5, Luckey3 [4, 12, 17]). Anaphylaxis represents the most ‘severe, life-threatening, generalized or systemic hypersensitivity reaction’ with multiple organs involved [31, 32], but scoring severity of an anaphylactic reaction in relation to exposure is complex due to the overall nature of anaphylaxis (progression, timing and interaction of symptoms), titrated challenges (terminated after the first clear objective signs) and treatment (immediately thereafter, hampering progression and overall severity). Therefore clear-cut anaphylactic reactions were identified and applied separately. Only 22% of the included challenges could by definition be classified as anaphylaxis [15], however all instruments included milder symptoms, such as urticaria (a grade 1–2 reaction) not reflecting life-threatening anaphylaxis [29]. Some instruments only cover the most severe anaphylactic reactions [11, 14], which is reflected in the translatability of milder reactions, while others are designed for the whole spectrum of reactions [5, 8], thereby addressing anaphylaxis and milder reactions similar.

We found a reverse causality between the numbers of steps in instruments and the percentage of all, non-anaphylactic challenges to be translated, meaning that fine-graded instruments were better in agreement with other tools concerning milder symptoms. All instruments could assess > 90% of the anaphylactic food challenges, whereas translatability for drug reactions are much more scattered; The explanation for this remains unclear, but as illustrated in Fig. 1, drug reactions manifest differently compared to food, with overrepresentation of non-anaphylactic skin symptoms. Surprisingly Ring/Messmer4 [10], developed for adverse colloid volume substitution reactions, scored milder than all other instruments applied on drug reactions, and further had reduced translatability on drug anaphylaxis compared to milder reactions. Some instruments entirely [10] or partly [11] focus on cardio-vascular rather than respiratory symptoms and signs, whereas others report that lethality, especially in children, is a result of respiratory compromise [33] or a combination of both [17]. This is mainly interfering with milder reactions and not with anaphylaxis and together with the differences in translatability indicates that fine-graded instruments mainly have their benefits among milder symptoms, whereas all instruments cope with anaphylaxis as “most severe”.

All instruments are organ-based, i.e. the skin, respiratory, gastro-intestinal, cardio-vascular, and nervous system, with symptoms classified into ordinal scales from 3 to 6 incomparable steps, ranging from “present” over “mild/moderate/severe” to the 6-step comprehensive Japanese ASCA-system [28]. Anaphylaxis after accidental exposure in non-controlled settings outside a hospital necessitates a relatively simple classification system easy to apply retrospectively. Classifying severity in terms of different grades (mild/moderate/severe) may be more informative for patients and non-allergy specialists, especially if reduced to a limited number of categories. However, for research purposes it may be more useful to have a numerical score of severity with more gradations. The overall/total severity of a reaction is then either based on the highest/most severe symptoms [7, 8, 10, 11, 14, 17, 21, 23–25], or calculated by different algorithms [6, 9, 12, 13, 20, 28–30]. Overall, instruments applying an algorithmic approach were neither superior in translatability nor distribution compared to Sampson5, with Zee3 [9] as only exception. However, a direct comparison of severity between most severe challenges revealed, that only half of grade 5 challenges in Sampson5 were translated into the most severe grade in Zee3, whereas milder reactions from multiple organs were converted into grade 3 in Zee3. Despite algorithms do not seem to add more information, iFAAM5 [5] is currently developing a comprehensive data-driven numerical scoring system (nFASS), which will be interesting to compare among existing instruments in relation to the balance between information gained and simplicity.

The retrospective application of instruments led to translational issues, where comparability and interpretation of known symptoms were critical, and especially type B (symptom missing in comparing instruments) and type D errors (Sampson5 contained less information than comparing instrument) caused discrepancies in the frequency of translation. Missing symptoms were an issue for all but one instrument [9], emphasizing the importance of a stable strategy to cope with these types of errors, which otherwise can lead to misclassification and thereby affect the overall severity of a reaction. In this study, symptoms not available were left untranslated, except for five instruments [2, 6, 10, 21, 29], where none-recorded ‘fainting’ dramatically would reduce the number of most severe anaphylaxis. One way to overcome missing specific symptoms are “catch-all” definitions [2, 4, 5, 9, 10, 12, 24, 30], i.e. all symptoms related to a specific organ, e.g. the “gastrointestinal tract”. Instruments including these have fewer type B arrows and thereby a higher translatability, in contrast to instruments with a predefined “symptom list”, which contains more information for research purposes, and avoids the pitfall of overseeing especially milder symptoms.

Skin symptoms usually include pruritus, urticaria, angioedema, flush/rash in 1–2 dichotomous outcomes. GI symptoms consist both of subjective symptoms (OAS, nausea, and abdominal pain) and objective signs (emesis and diarrhea). Brown(A)3 [17] found a direct link between GI symptoms and hypotensive anaphylaxis, whereas Niggemann6 [29] claims that GI symptoms are over-represented, which is reflected in Niggemann6 being milder compared to Samspon5 both after food, where GI symptoms are expectedly predominant, but surprisingly also after drug challenges. Cardio-vascular symptoms are characterized by a change in heart rate (from tachycardia to cardiac arrest) and degrees of hypotension, where only few instruments have an exact definition [11, 17, 18, 25, 28]. Neurological symptoms are less consistent with grades of anxiety and consciousness (from reduced activity level to total loss of consciousness). Niggemann6 [29] claims that subjective symptoms such as anxiety, malaise, weakness or dizziness should not form the basis for grading an allergic reaction, however 70% (77/110) of our challenges with neurological subjective symptoms also have clear-cut objective signs from other organs. Terminating a challenge based on neurological symptoms is therefore rare and can be avoided by strict clinical stop-criteria. The biggest discrepancies are found in respiratory symptoms; some instruments only apply airway obstruction (defined as asthma, cyanosis, or respiratory arrest [8]), symptoms from upper airways, i.e. nose and from eyes are covered by some [8, 20, 25] and are excluded by others [10, 13, 17]. The interpretation of the respiratory system as one system including nose, pharynx, larynx, and bronchial is lacking, and especially symptoms from tongue and pharynx are vaguely mentioned. The compression of ‘cough, hoarseness, dysphagia’, and ‘wheezing, asthma, dyspnea, cyanosis’ into two overall ‘laryngeal’ and ‘bronchial’ categories, and the lack of ‘stridor’, a seldom but adrenalin-requiring laryngeal symptom, hamper Sampson5 [8], which have now prompted a change of in our department to facilitate this.

The incomplete translatability and the different number of steps among the instruments make the severity distribution difficult to compare. No standardized or validated method exists to compare multiple heterogeneous scoring systems; some instruments (iFAAM5, Niggemann6, ASCA6, SFFA4, Ring-Behrend4, NewEAACI3 [5, 21, 23, 28–30] have high translatability i.e. percentage translated while others have a similar distribution of severity (Brown(B)3 [18]). The paired kappa comparison probably does not reflect the situation, where two clinical settings intend to compare severity on two different populations, but it is the methodologically correct way to asses this in our retrospective study. By applying the CDF-curve, we assumed that severity obtained under standard challenge conditions was normally (Gaussian) distributed. A linear relationship between the grades, i.e. fixed and equal distance between steps, is also assumed but hypothetical.

The simplified distribution of instruments in reference to Sampson5 place them into 3 categories; milder, similar or more severe than Sampson5. Sampson5 was original applied at our sitting for historical reasons, mainly due to the high numbers of pediatric food challenges performed in our clinic. We do not claim that any of the instruments is better or worse to score severity of anaphylaxis, nor that Sampson5 is the gold standard. This simply identifies the difference between instruments, which reflects their heterogeneous etiology, and should be considered when comparing existing scoring systems for severity in anaphylaxis. This also emphasize, that instruments applied beyond their initial purpose have limitations, especially embracing milder reactions, and might reflect altered distribution of severity.

## Conclusion

We found a reverse causality between the numbers of grades an instrument span and the percentage of non-anaphylactic challenges to be translated, whereas anaphylaxis more easily is translated between instruments. The distributions in severity were different; some over-estimate e.g. having more severe reactions than Sampson5 [21, 30], whereas others under-estimate [10, 13, 25]. There is no consistency between food and drug challenge severity distribution; some scored food challenges more severe than drug challenges [5, 7] and others drug challenges more severe than food [4, 12, 17]. Most instruments appraise milder symptoms identical to anaphylaxis, whereas few weighted them more [14, 17, 20, 27] or less severe [13]. Instruments developed to cope with hymenopteran reactions [11–14] overall had poor translatability and distributed differently compared to Sampson5. Drug challenges are complicated to compare [10], and finally algorithms do not add more information, but compromise comparison of especially milder symptoms.

## Abbreviations

ASCA: Anaphylaxis Scoring Aichi
AUC: area under curve
CDF: cumulative distribution function
CT: clinical trials
CV: cardio-vascular
DSA: Danish Society for Allergology
EAACI: European Academy of Allergy and Clinical Immunology
ER: emergency rooms
GI: gastro-intestinal
ICU: intensive care units
iFAAM: integrated approaches to food allergen and allergy risk management
nFASS: numerical food allergy severity system
NSAID: non-steroidal, anti-inflammatory, drug
OAS: oral allergy syndrome
oFASS: observational food allergy severity system
ORCA: Odense Research Center for Anaphylaxis
SFFA: Svenska Föreningen För Allergologi (Swedish Association for Allergology)
